# THE ROLE OF GERONTOLOGISTS IN ACHIEVING THE UN’S SUSTAINABLE DEVELOPMENT GOALS

**DOI:** 10.1093/geroni/igz038.293

**Published:** 2019-11-08

**Authors:** Toni C Antonucci

**Affiliations:** University of Michigan, Ann Arbor, Michigan, United States

## Abstract

The Sustainable Development Goals (SDG’s) developed by the United Nations in 2015 have as their underlying theme, the pledge that no one will be left behind. The SDGs address global poverty, inequality, climate change, the environment, peace and justice. They are intended to be global benchmarks to be reached by 2030 to ensure well-being and prosperity while protecting and promoting human rights and freedoms (UN, 2015). They envision a world without poverty, where all persons can live with dignity and security in societies free of violence and discrimination based on the foundation of universal human rights. The 17 goals and their 169 targets cover a range of social and economic development issues from poverty and gender inequality to climate change and sustainable cities. These goals are both interrelated and indivisible with each important for individual and social well-being. For example, achieving gender equality can help eradicate poverty while improved health can contribute to increased individual productivity and economic growth. Unfortunately, the needs of older people are larger ignored. In this symposium we outline how gerontologists can and have contributed to the SDG goals. We provide input from four disciplines whose work directly addresses the needs of older people. The four disciplines are: health- how do we meet the health needs of older people, psychology – what are the mental health issues facing older people, public policy – how can\has governments assist through laws and policy, and social work – how can social work address the needs of the vulnerable old.

